# Variable and conserved B cell epitopes of GII.4 human noroviruses

**DOI:** 10.1128/jvi.01804-25

**Published:** 2026-01-13

**Authors:** Gabriel I. Parra, Lauren A. Ford-Siltz, Kelsey A. Pilewski, Kentaro Tohma, Michael Landivar, Joseph A. Kendra

**Affiliations:** 1 Division of Viral Products, Center for Biologics Evaluation and Research, Food and Drug Administration 4137 , Silver Spring, Maryland, USA; Indiana University Bloomington, Bloomington, Indiana, USA

**Keywords:** noroviruses, calicivirus, antigenic variation, vaccines, gastrointestinal infection

## Abstract

Norovirus is a leading cause of acute gastroenteritis worldwide, imposing a major burden on public health and healthcare systems. Despite its significant medical impact, no licensed vaccines or specific antiviral therapies are currently available. Norovirus vaccine development is complicated by several factors, including the extreme genetic and antigenic diversity. In particular, the predominant genotype GII.4 exhibits a continuous emergence of novel variants that can evade immune responses acquired from previous infections. In this manuscript, we will summarize the characteristics and current knowledge of the B cell responses elicited by conserved and variable GII.4 epitopes and discuss how these findings inform our understanding of responses to other pandemic norovirus genotypes. We also highlight how ongoing research in this area may provide critical insights for the development of broadly protective norovirus vaccines.

## INTRODUCTION

Human noroviruses are the leading viral cause of acute gastroenteritis worldwide. Infections are spread through fecal-oral transmission following exposure to contaminated food or infected individuals, producing symptoms of abdominal cramping, vomiting, and diarrhea within 12–48 hours. Norovirus disease is typically quick to resolve in healthy individuals but can pose a risk for prolonged or life-threatening complications in children, the elderly, or immunocompromised individuals (1). Globally, norovirus disease accounts for approximately 200,000 annual deaths (primarily in developing countries) and imposes an annual financial burden of approximately $60 billion from productivity loss and health care expenses (2, 3).

**Fig 1:**
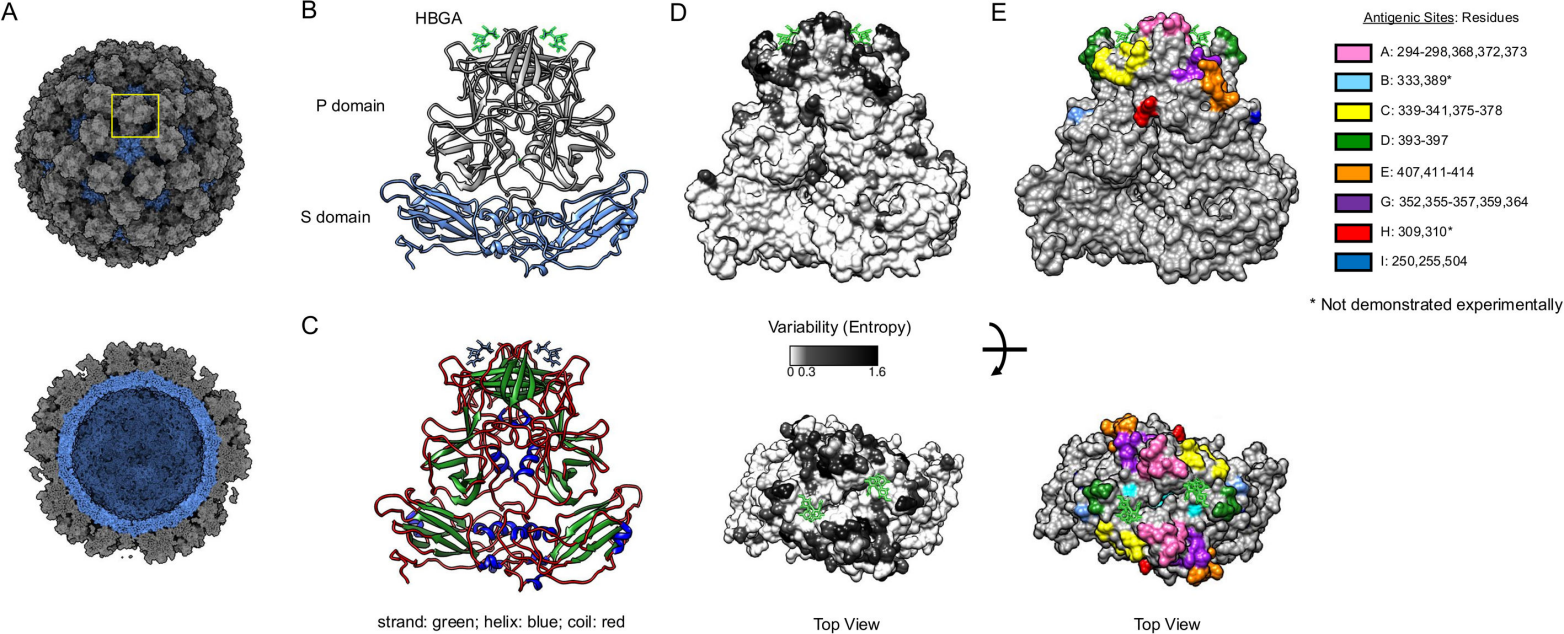
Depiction of norovirus major capsid protein, VP1. (**A**) Model of the norovirus virion. One VP1 dimer is indicated within a square. (**B**) Representation of the VP1 dimer showing the two structural domains, P and S. The P domain is subdivided into P1 and P2 domains. Same representation of the VP1 dimer showing the secondary structures (**C**), the variability (**D**), and antigenic sites (**E**). Variability was calculated using 500 (from a total of 3,108) sequences randomly selected (50/variant) from viruses circulating during 1995–2022 and Shannon Entropy (13, 14). The HBGAs are shown as sticks. The structural model of the VP1 dimer was acquired from the Protein Database (PDB: 7K6V), and the figures were rendered using UCSF Chimera (15, 16).

The human norovirus genome is a single-stranded, positive-sense RNA that is organized into three open reading frames (ORFs). The first ORF encodes a polyprotein that is co-translationally cleaved into six nonstructural proteins. ORFs 2 and 3 encode the major (VP1) and minor (VP2) structural proteins, respectively (4). The norovirus virion is composed primarily of an array of VP1 capsid proteins assembled into an icosahedral particle (Fig. 1A). Each VP1 consists of a shell (S) domain and a protruding (P) domain, which are connected by a flexible hinge region (Fig. 1B). The S domain has a classical jelly roll (β-strand sandwich) motif associated with viral capsids, while the P domain presents a unique structure. The P domain is divided into two subdomains, P1 and P2. The outermost P2 subdomain folds into a barrel-like structure consisting of six β-strands, which is located on top of the P1 subdomain (5). While VP1 presents multiple secondary structures, ~65% of the residues form coils that connect these secondary structures (Fig. 1C). Although multiple epitopes and antibodies mapped on nonstructural proteins have been identified (6, 7), most of the humoral responses to noroviruses are directed to the VP1 with neutralizing antibodies mapping to the P domain (8, 9). Moreover, strong binding interactions have been identified between the P2 subdomain and histo-blood group antigens (HBGA), which are cellular ligands that facilitate norovirus infection (10–12).

Immunological research on noroviruses has long been constrained by the inability to propagate these viruses in traditional cell culture systems. To overcome this limitation, the prevailing approach has been the expression of the VP1 protein from norovirus strains of interest, which self-assembles into virus-like particles (VLPs) that can be purified for study. These genetically inert VLPs antigenically resemble wild-type virions and exhibit binding interactions to HBGAs (17), thereby making them indispensable tools for immunoassays and vaccine development. Indeed, studies using VLPs have shown that antibodies blocking the interaction of norovirus with HBGAs correlate with disease protection in humans (18, 19). In recent years, a human intestinal enteroids system has been developed that supports the cultivation of certain strains of human noroviruses (12, 20, 21), enabling measurement of antibody-mediated norovirus neutralization and validation of the HBGA blocking assay as a surrogate of norovirus neutralization (8, 9, 22, 23).

Noroviruses are broadly classified into 10 genogroups (GI through GX) and further subdivided into genotypes and variants based on sequence differences arising on the VP1. Norovirus diversification and classification also extend to the nonstructural proteins, with more than 40 polymerase types identified in human noroviruses based on their genetic diversity on RNA-dependent RNA polymerase sequences. Recombination events mostly occur at the junction between ORF1 and ORF2, resulting in viruses that possess distinct combinations of capsid and polymerase types. An analysis of the global distribution of noroviruses between the years 1995 through 2019 identified 37 genotypes—primarily from GI and GII genogroups—that are capable of infecting humans. However, the GII.4 noroviruses are notably predominant compared to the other genotypes, accounting for ≥50% of all reported infections worldwide (24, 25). The predominance of GII.4 circulation has been mainly attributed to the continuous chronological emergence, proliferation, and replacement of antigenically distinct variants that can escape herd immunity acquired from previous infections (13, 26–28). The determinants for these antigenic changes were mapped to mutations on the P2 subdomain that change in synchrony during the diversification of a new variant (9, 13, 29–31). Several pandemic GII.4 variants have emerged over the past 30 years: Grimsby_1995, FarmingtonHills_2002, Hunter_2004, Yerseke_2006a, DenHaag_2006b, NewOrleans_2009, and Sydney_2012. Other GII.4 variants have also been identified during this time: Sakai_2003, Osaka_2007, Apeldoorn_2007, and Hong Kong_2019, whose influence has largely been relegated to regional outbreaks. Recombination events can also be drivers of emerging GII.4 viruses, as evidenced by the global surge of GII.4 (e.g., GII.4[P12], GII.4[P16], and GII.4[P31]) with distinct nonstructural proteins (25).

Major advances have been made over the past decade toward our understanding of immunity and the antigenic diversification of noroviruses. In this review, we will summarize the current knowledge of the conserved and variable B cell epitopes described for GII.4 noroviruses and discuss how further characterization of the antibody responses to norovirus infection and vaccination can inform efforts to develop vaccines and therapeutics to these fast-evolving viruses.

## GII.4 VARIABLE EPITOPES

The intrinsic structural nature of VP1 seems to facilitate norovirus diversification, with >80% of the variable sites from GII.4 human noroviruses mapping on coils. Most of this variation is present in the P domain (Fig. 1D), which has been shown to be the primary target of the humoral responses (9). GII.4 noroviruses present five major variable antigenic sites (A, C, D, E, and G, Fig. 1E), whose mutational profile has been associated with the emergence of variants that cause concurrent outbreaks globally (9, 13, 14, 32, 33). The residues forming these antigenic sites were deduced from the variability presented by the different variants that emerged since the 1990s and from the binding profile of mutant VLPs to mouse and human monoclonal antibodies (mAbs) (9, 13, 27–30, 33, 34). Notably, these antigenic sites include multiple different epitopes that overlap around these variable residues on the surface of the viral capsid (9, 35, 36). There are three additional small variable motifs (B, H, and I) that map on the surface of VP1 (Fig. 1E). While some broadly reactive mAbs were mapped to site I (9, 36), no mAb has yet been characterized for motifs B or H, so their role on the antigenic topology of GII.4 noroviruses remains to be determined. Based on structural information of mAbs mapping to conserved regions of the P domain, Lindesmith and colleagues have classified residues mapping to antigenic site I into two different antigenic sites: F (254–257, 327, 404) and I (402, 403, 504–506) (36); however, it is worth noting that only three residues (250, 255, and 504) showed variability within this region, and a mAb mapping to all three variable residues has been identified, suggesting that they are part of a single epitope (9). All these variable antigenic sites are adjacent to the conserved HBGA binding pocket (Fig. 1E). Thus, it is thought that antibodies binding to these antigenic sites neutralize virus infection by sterically blocking viral interaction with the ligands and potentially with the unidentified cellular receptor (37). Of note, as the VP1 protein length among human-infecting genotypes varies between 530 and 550 residues, extrapolating GII.4 epitopes to other genotypes should be approached with detailed structural analysis.

### Antigenic site A

**Fig 2:**
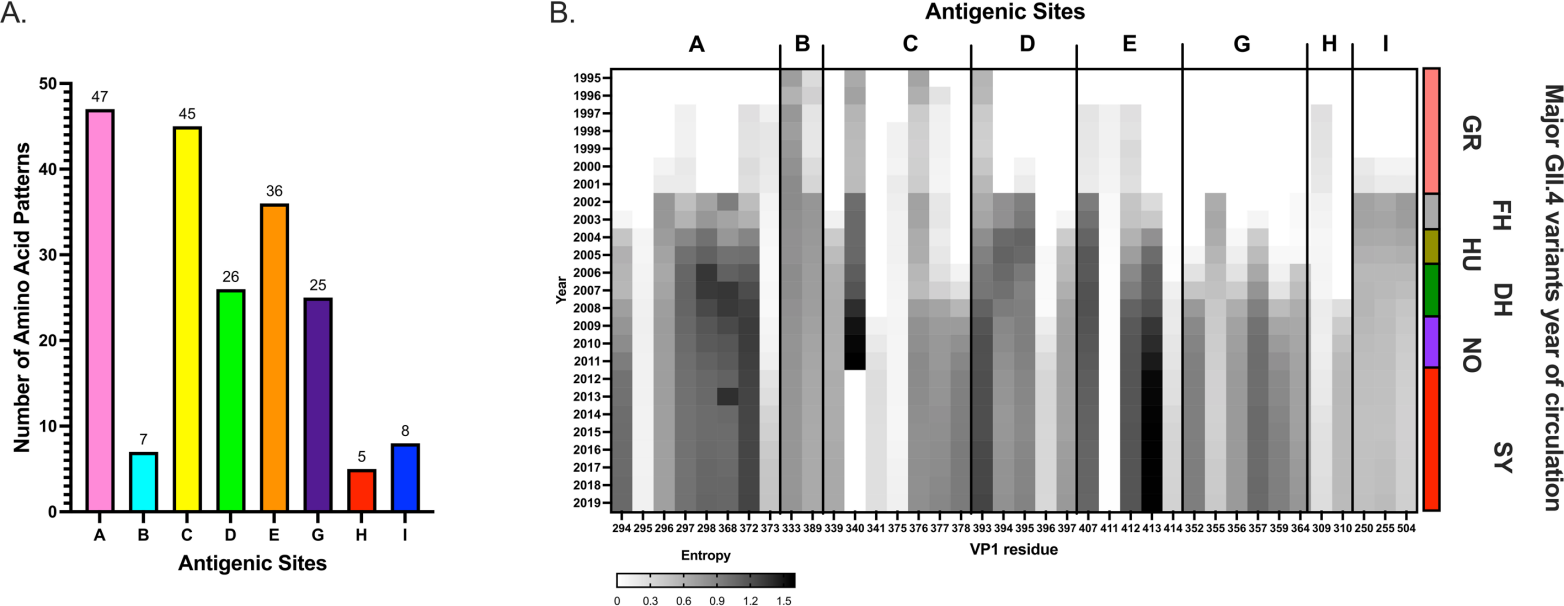
Genetic diversification of GII.4 antigenic sites. (**A**) Variability patterns of GII.4 antigenic sites. Patterns were calculated using amino acid combinations from 3,108 VP1 sequences of GII.4 noroviruses as implemented elsewhere (13, 14). Only amino acid combinations presented in >2 viral sequences were included in the analysis. (**B**) Temporal diversification of amino acids mapping on GII.4 antigenic sites. Shannon entropy values were calculated for each residue in a cumulative fashion. The data set included a subset of randomly sampled (50 per variant) sequences of VP1 sequences of GII.4 noroviruses as implemented in Fig. 1D. Variant abbreviations are as follows: GR: Grimsby_1995; FH: FarmingtonHills_2002; HU: Hunter_2004; DH: DenHaag_2006b; and NO: NewOrleans_2009; SY: Sydney_2012.

This antigenic site is located at the apex of the VP1 protein and formed by residues from two coils (residues: 294–298, 368, 372, and 373), which are close to the HBGA binding site. This site is the largest antigenic site and is composed of eight residues with high variability and therefore presents the largest number of potential mutational combinations (Fig. 2A) (38). It has been identified as one of the most immunodominant blocking antigenic sites (9, 39), and it was the first to be determined to play a role in the emergence of new GII.4 variants (33). Despite its broad diversity, multiple broadly neutralizing mAbs have been described mapping to this site. Most cross-reactivity patterns seem to be associated with those that bind ancestral (Camberwell_1987, Grimsby_1995, FarmingtonHills_2002, and Hunter_2004) or contemporary (DenHaag_2006b, NewOrleans_2009, and Sydney_2012) variants (28, 29, 38, 40–42). The latter is in line with results using polyclonal sera from VLP-immunized mice (24). Exceptions to this cross-reactive pattern have been reported: (i) a mouse mAb developed against DenHaag_2006b VLPs (GII.4-2006-G3), presented blocking activity against the FarmingtonHills_2002, DenHaag_2006b, and NewOrleans_2009 variants, but not against Camberwell_1987, Grimsby_1995, or Sydney_2012 (40, 43), (ii) human mAbs that recognize (and block HBGA interaction of) two viruses as temporally distant as Grimsby_1995 and Sydney_2012 but not many of the intermediate variants (42)*,* and (iii) human and mouse mAbs that were uniquely specific to the viruses from a single variant (9, 14, 28, 29, 34, 41, 42). Notably, a human mAb (NORO-123) that was developed from an individual infected with a Sydney_2012 virus was mapped to the variable antigenic site A and has shown strong HBGA-blocking titers to all strains tested spanning 25 years of GII.4 circulation (42). Several additional data have shown that cross-reactive neutralizing antibodies mapping to antigenic site A can be elicited during infection and vaccination (28, 36, 42, 44). The binding and competition patterns of mAbs mapping to this antigenic site suggest that it is made of multiple overlapping epitopes that anchor to only some of the variable residues (22, 34). The latter seems to play an important factor for the elicitation of broadly neutralizing antibodies to this antigenic site, so further structural analyses are warranted to determine how B cells recognize this immunodominant antigenic site. Finally, the introduction of mutations in this antigenic site has been shown to ablate HBGA binding (9); thus, despite not being part of the HBGA binding pocket, this suggests that GII.4 noroviruses need to balance antigenic changes with HBGA binding properties. Indeed, although this antigenic site exhibits the greatest diversity in possible amino acid combinations (Fig. 2), a complete reversion to an epitope composed of co-evolving residues 297 and 372—originally described in the Grimsby_1995 and FarmingtonHills_2002 strains—was observed during the antigenic evolution of the Sydney_2012 viruses (14) (Fig. S1).

### Antigenic site C

This antigenic site is located on the lateral side of the P domain and encompasses residues that map to two coils (residues: 339–341, 375–378) that are close together in the tertiary structure. Contrary to antigenic site A, this antigenic site has shown a gradual increment in the number of variable sites during the diversification of GII.4 noroviruses (Fig. 2). Thus, during the transition of Grimsby_1995 to FarmingtonHills_2002, only two residues (E340G and E376Q) present mutations, and during the transition of FarmingtonHills_2002 to Sakai_2003 and DenHaag_2006b, two additional residues (K339R and G378H) started to present prevalent mutations (Fig. 2). An additional new mutation (D341N) was detected with the emergence of the NewOrleans_2009 variant, which reverted to the original residue (N341D) in Sydney_2012. Despite the mutational profile showing a direct correlation with the emergence of GII.4 variants (13), this antigenic site seems to play only a minor role in the context of polyclonal responses for two of the variants tested for overall immunodominance of the variable sites (9). Because of their proximity to antigenic site A, C-binding mAbs have been shown to block A-binding mAbs in competition assays (13, 34); however, the reciprocal has not been observed. One human mAb mapping to this antigenic site has been shown to cross-react to multiple different variants (42), so the role of antibodies mapping to this site on the overall cross-reactive responses should not be understated.

### Antigenic site D

**Fig 3:**
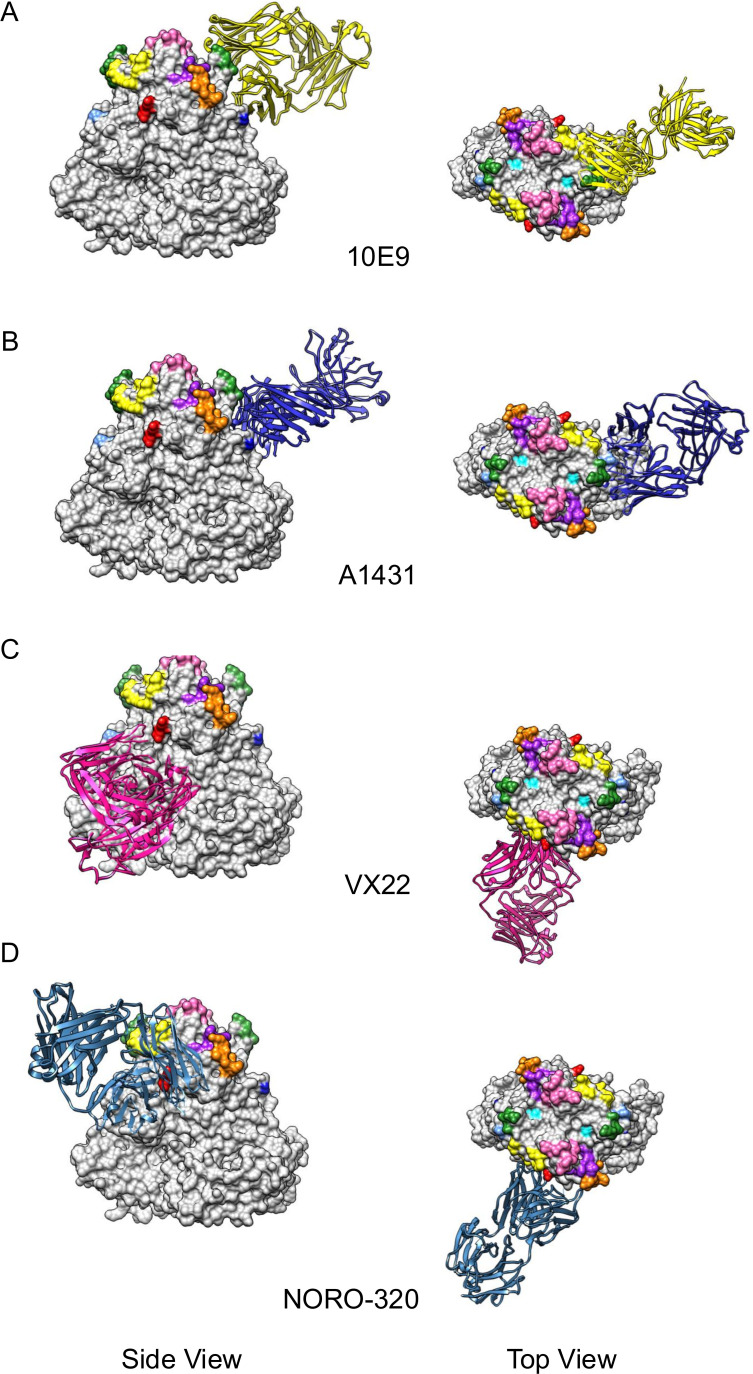
GII.4 norovirus VP1 structures in complex with different neutralizing mAb fragments. (**A**) Mouse neutralizing antibody 10E9 (PDB: 6EWB) binding to antigenic sites C and D (35). (**B**) Human neutralizing antibody A1431 (PDB: 6N8D) binding to conserved antigenic site I. (**C**) VX22 (PDB: 8VKX) and (**D**) NORO-320 (PDB: 7JIE) bind to a highly conserved region of the P1–P2 interface (36, 48). The different mAb fragments are shown in ribbon, and the GII.4 antigenic sites are shown using the same colors as in Fig. 1. The structural model of norovirus VP1 dimer was acquired from the Protein Database (PDB: 7K6V), and the figures were rendered using UCSF Chimera (15, 16).

This antigenic site is located on the opposite side of antigenic site C, but the dimeric nature of VP1 brings these two antigenic sites very close to each other (Fig. 1E and 3A). Indeed, one of the few structures available for neutralizing antibodies, mAb 10E9, demonstrated that the epitope includes residues from these two antigenic sites (Fig. 3A). Antigenic site D includes residues from a single coil (residues: 393–398), but despite their linear nature, all antibodies described recognize conformational epitopes (9, 28, 35, 45), suggesting that additional (nearby) conserved residues form the epitopes from these antibodies. Indeed, X-ray analyses demonstrated that the footprint of mAb 10E9 included several highly conserved residues of the P domain (Fig. 3A) (35). Antibodies mapping to this antigenic site have been shown to play an important part of the overall polyclonal responses to FarmingtonHills_2002 viruses, but not to Sydney_2012 viruses (9). An amino acid insertion after residue 392 was associated with the emergence of the FarmingtonHills_2002 variant (46). This insertion has been present in all major viruses circulating since its emergence in 2002 (9). Viral sequences without this insertion were detected in populations from immunocompromised patients in the US (45, 47), but those were not detected on a global scale. It is possible that this insertion changed the overall antigenicity and redirected the immunodominance to site A during the emergence of FarmingtonHills_2002 viruses (9), the ancestral variant of all contemporary viruses. It has also been shown that changes on this antigenic site modulate viral binding to HBGAs (29, 32, 45). Additional studies that quantitatively evaluate the role of this insertion in HBGA binding and the overall antigenic profile of GII.4 viruses are still needed to better understand the role of this (and other) insertions detected in GII.4 viruses.

### Antigenic site E

Of all the variable antigenic sites, antigenic site E is the most distal from the HBGA carbohydrate binding pocket (Fig. 1E). Similar to antigenic site D, this antigenic site has been shown to play an important role in the overall polyclonal blocking responses to FarmingtonHills_2002 viruses, but not to Sydney_2012 (9, 30). This site includes residues from a single coil (407–414), but only half of these residues (i.e., 407, 412–414) are variable. Despite mapping on the surface, residues 410 and 411 show a high degree of conservation, suggesting a potentially important role in maintaining the structural integrity of the capsid protein or the interaction with cellular factors. Although only a few mAbs mapping to this site have been identified (9, 30), a large subset of mAbs binding to antigenic sites E and G (see next subsection) has been identified in mice immunized with a FarmingtonHills_2002 strain (9). Because mutations in either E or G alone do not abrogate binding, their role in the overall immune response might require concurrent mutations on those two sites to result in a significant antigenic diversification (31).

### Antigenic site G

This antigenic site was recently described using all publicly available complete VP1 sequences (9, 13). This site includes six variable residues (352, 355–357, 359, and 364) from a single coil, but it was overlooked as it showed a remarkable conservation among viruses circulating prior to 2006 (Fig. 2). This site is centrally located between antigenic sites A, D, and E (Fig. 1E); thus, it is unsurprising that the large majority of G-mapping mAbs present epitopes with residues mapping to these antigenic sites (9, 41). Notably, a striking difference in the immunodominance role of this antigenic site was detected between FarmingtonHills_2002 and Sydney_2012 variants. This difference resulted in the gradual change of the immunodominance of antigenic site A to G during the period of rapid turnover of variants (2002–2012), which was associated with the mutation S352Y (polar uncharged to hydrophobic) (9). Consistent with the mutational pattern of residue 352, mice and human mAbs binding to site G have shown cross-reactive patterns that include either ancestral variants (Camberwell_1987, Grimsby_1995, and FarmingtonHills_2002) or contemporary variants (DenHaag_2006b, NewOrleans_2009, and Sydney_2012) (9, 36). The reasons for this change of immunodominance are not fully understood, but the two GII.4 variants that presented the longest circulation in humans (Grimsby_1995 and Sydney_2012) showed a strong immunodominance of antigenic site G. On the contrary, those with immunodominant antigenic site A present a rapid turnover due to the focused, strong immune response against a single variable site, an event seen in H3N2 influenza viruses (49). A better understanding of the immunodominance of each variable antigenic site across different variant backgrounds could provide essential insights for the development of cross-protective vaccine candidates.

## INTERACTION OF VARIABLE ANTIGENIC SITES AND VARIANT EMERGENCE

**Fig 4:**
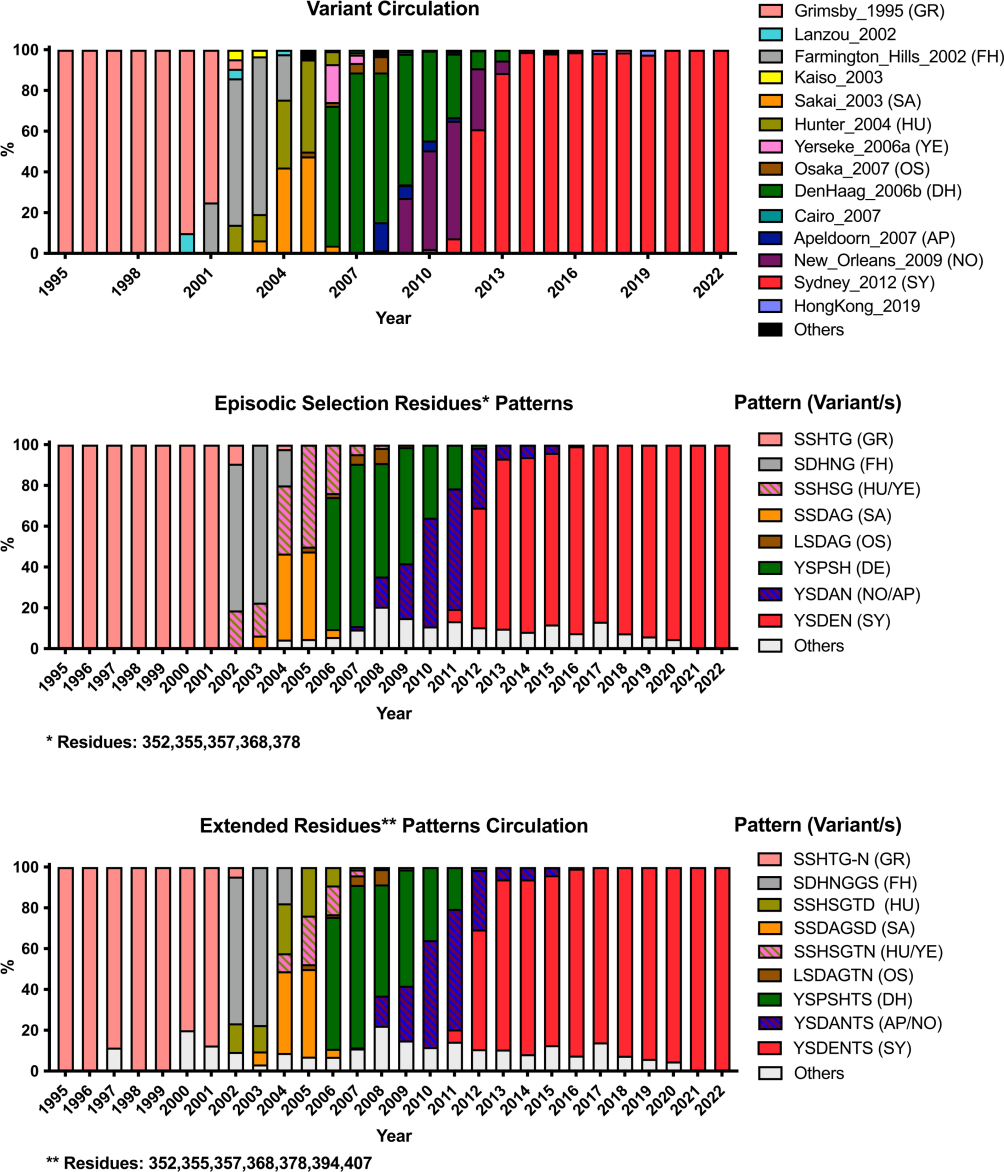
Temporal distribution of the variants and amino acid patterns on GII.4 noroviruses. GII.4 variant yearly distribution tabulated using 3108 VP1 sequences (14). Color of the bars corresponds to the most frequent sequence patterns presented at variants. Variant abbreviations are as follows: GR: Grimsby_1995; FH: FarmingtonHills_2002; HU: Hunter_2004; DH: DenHaag_2006b; NO: NewOrleans_2009; SY: Sydney_2012; SA: Sakai_2003; YE: Yerseke_2006a; OS: Osaka_2007; and AP: Apeldoorn_2007. Analyses were conducted using scripts implemented elsewhere (13, 14).

The emergence of different GII.4 variants is characterized by changes on the variable antigenic sites (13, 27). Indeed, an analysis of all VP1 sequences available demonstrated that episodic diversification of these variants was associated with codons encoding five residues (352, 355, 357, 368, and 378) that map to the variable antigenic sites (13). Moreover, patterns of co-evolution were identified with additional residues making the antigenic sites (e.g., 297/372, 357/372, 357/397, and 395/504), suggesting a strong interaction among these variable antigenic sites during the evolution of GII.4 noroviruses (31). Thus, while the “core” residues involved in the emergence of GII.4 variants seem to be evolving under positive selection (352, 355, 357, 368, and 378), additional residues mapping to other antigenic sites (e.g., 394 and 407) seem to follow the same mutational profiles associated with the emergence of new antigenic variants (Fig. 4). These patterns of diversification can be linked to the fact that these epitopes are close on the tertiary and quaternary structure of the VP1 dimers (Fig. 3), so the epitope of several antibodies maps to two or more antigenic sites in the capsid of GII.4 norovirus (9). Thus, immune pressure leads to concurrent changes in multiple antigenic sites for the emergence of antigenically distinct variants (31).

## CONSERVED EPITOPES

Although different degrees of cross-reactivity have been described for antibodies mapping to the conserved S domain and variable P domain, most of them fail to show cross-neutralization activity to all GII.4 variants/viruses described for the last four decades (50). Antibodies binding to the highly conserved P1/P2 interface have been described, and their epitopes have been mapped with structural techniques. The first structural characterization of such antibodies includes that for broadly reactive mAb against GII.4, GII.10, and GII.12 viruses, 5B18, which showed a footprint that included residues from the C terminal part of VP1 (GII.10: 433, 496, 530, 533, 534, and 535) (51). This antibody was not tested for HBGA blocking (or viral neutralizing) activity, and its binding structurally clashes with the S domain (Fig. S2A). How this antibody is able to bind intact particles remains unclear (51). Using antibody repertoire analyses, two human cross-reactive mAbs were detected after immunization with the bivalent GII.4c/GI.1 VLPs (41). The first one, A1227, showed broad reactivity to GI and GII norovirus but not HBGA blocking activity. A1227 showed a different footprint from mAb 5B18 (Fig. S2A and B), demonstrating that different regions of the P domain elicit broadly reactive antibodies. Notably, despite having minor clashes with the S domain (41), the A1227 antibody was not able to bind particles engineered to prevent dissociation into VP1 monomers or multimers (52). The second antibody, A1431, showed blocking activity against numerous GII.4 strains spanning from 1987 to 2015 (41). The critical residues involved in A1431 binding were determined to be: 402, 403, 504, and 506 (Fig. 3B), with only residue 504 exhibiting genetic variability (Fig. S3). While these residues are conserved in contemporary strains, they showed variation in Grimsby_1995, FarmingtonHills_2002, and early viruses from DenHaag_2006b (Fig. S3) (13). The broad reactivity presented by A1431 suggests that none of these mutations affected its blocking activity to viruses circulating over the past four decades (41). Given that A1431 accounted for only ~10% of the overall response following immune boosting with vaccine candidates, and that experimental immunization of animals with VLPs designed to focus responses on conserved regions failed to elicit cross-blocking responses in serum (53), further studies are warranted to explore strategies to enhance the immunodominance of antibodies mapping these conserved regions of the viral capsid protein. Finally, the presence of broadly reactive and neutralizing human antibodies against multiple GII genotypes (NORO-320 and VX22) that bind to a highly conserved region that includes residues from P1 and P2 subdomains has been demonstrated in humans (Fig. 3C and D), providing hopes for the development of broadly protective vaccines for GII noroviruses (36, 48). Structural characterization of these broadly reactive (NORO-320 and VX22) antibodies suggests that they do not compete for the same epitope or structural space (Fig. 3C and D and Fig. S2C and D). However, non-blocking antibodies (A1227) seem to compete for the same structural space of broadly neutralizing (VX22) antibodies (Fig. 3C and Fig. S2B and C), which could limit the overall immune effect. Future studies on the competition patterns and structural occupancy of the distinct antibodies elicited during natural infection and vaccination are required for a holistic comprehension of the protective role of humoral responses.

## CONCLUDING REMARKS

Significant progress has been made over the past decade in our understanding of GII.4 norovirus evolution and immunity. The binding profiles of nearly 200 mAbs have been characterized, offering valuable insights into the immunological pressures that drive the emergence of new GII.4 variants. Despite these advances, a comprehensive mapping of B cell epitopes—including the identification of conserved residues flanking variable ones—and a full understanding of how these antibodies interact with the intact virion remain outstanding challenges. We also now know that the mutational profiles of some of these epitopes include reversions that recapitulate ancestral epitopes on current viruses (14), suggesting constraints on the evolvability of these antigenic sites. Finally, the application of machine learning techniques has demonstrated the ability to identify key residues involved in the antigenic diversification of GII.17 variants (54), thereby opening new avenues to accelerate our understanding and predict models of norovirus antigenic evolution. A deeper understanding of the principles governing these mutational patterns, their effects on antibody recognition, the role of antibody interaction in the context of the whole virion, and the role of broadly neutralizing antibodies in shaping immune responses will be crucial for guiding vaccine development against the continually evolving norovirus.

## SUPPLEMENTAL MATERIAL

Fig. S1 jvi.01804-25-s0001.pdf Variability pattern of residues 297 and 372 of the GII.4 major capsid protein, VP1.

Fig. S2 jvi.01804-25-s0002.pdf GII.4 norovirus VP1 structures in complex with different broadly reactive monoclonal antibodies.

Fig. S3 jvi.01804-25-s0003.pdf Variability pattern of residues 402, 403, 504, and 506 of the GII.4 major capsid protein, VP1.

Supplemental legends jvi.01804-25-s0004.docx Legends for Fig. S1 to S3.

ASM does not own the copyrights to Supplemental Material that may be linked to, or accessed through, an article. The authors have granted ASM a non-exclusive, world-wide license to publish the Supplemental Material files. Please contact the corresponding author directly for reuse.
