# Supplementary figures and images for: Variable and conserved B cell epitopes of GII.4 human noroviruses
Source: J Virol. 2026 Jan 13;100(2):e01804-25. doi: 10.1128/jvi.01804-25 (PMC12911863; doi:10.1128/jvi.01804-25)

Figure S1

Mutational Pattern Residues:  
297, 372

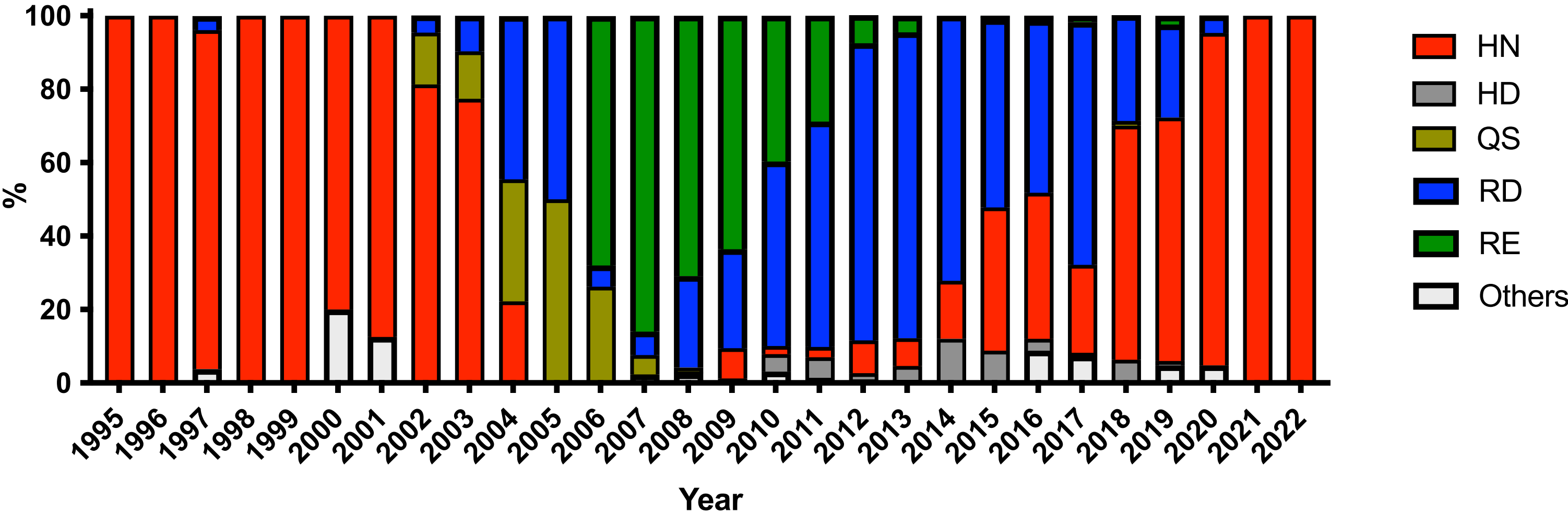

Supplement: Fig. S1 — Variability pattern of residues 297 and 372 of the GII.4 major capsid protein, VP1. [file jvi.01804-25-s0001.pdf]

Figure S2

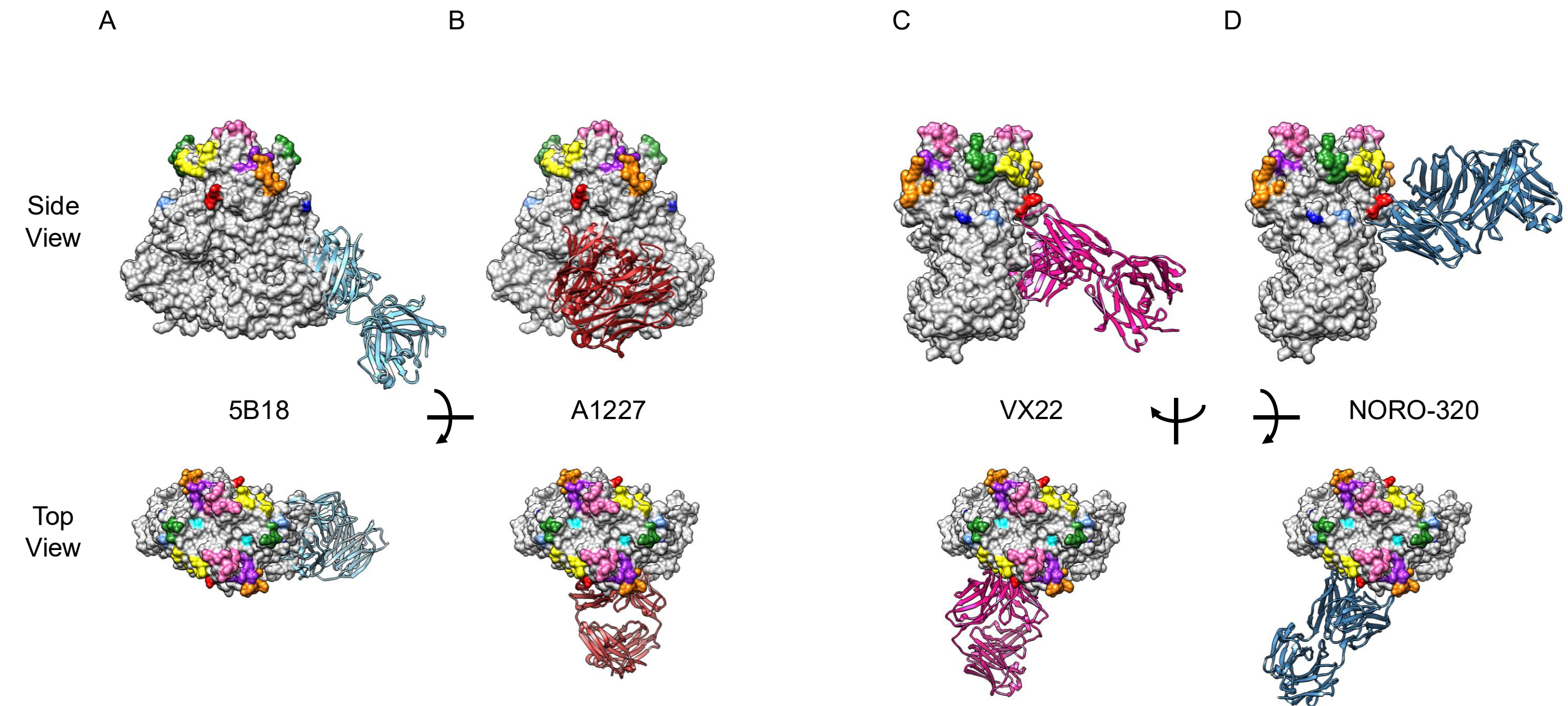

Supplement: Fig. S2 — GII.4 norovirus VP1 structures in complex with different broadly reactive monoclonal antibodies. [file jvi.01804-25-s0002.pdf]

Figure S3

**Mutational Pattern Residues:  
404, 405, 504, 506**

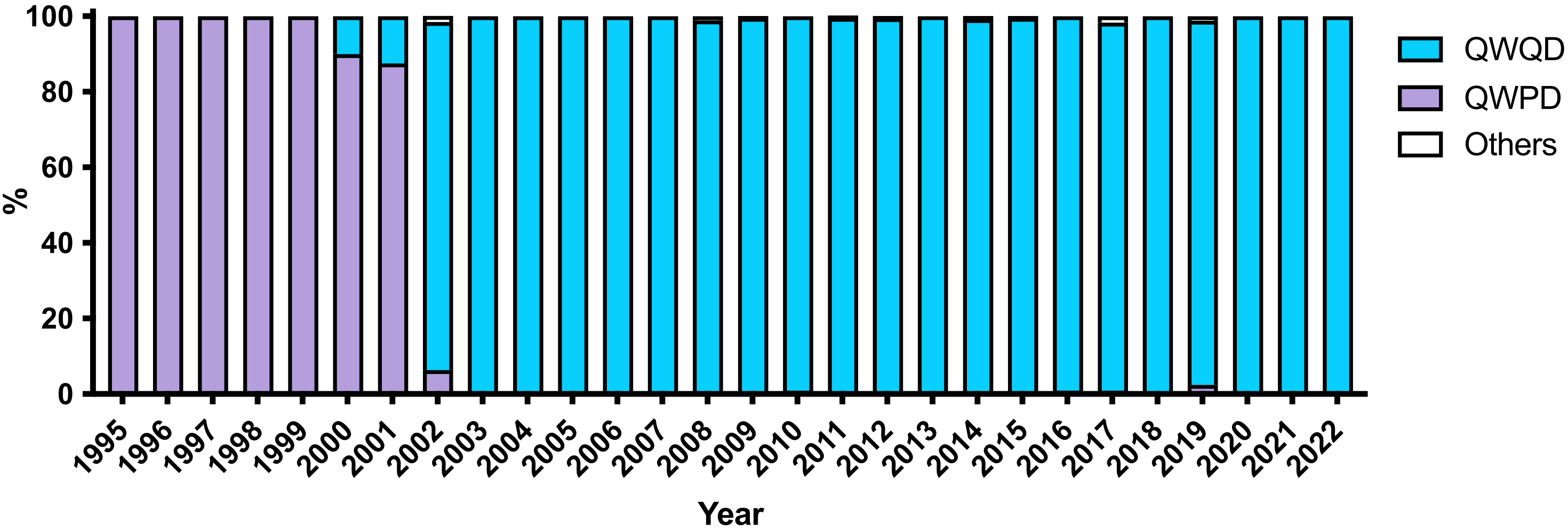

Supplement: Fig. S3 — Variability pattern of residues 402, 403, 504, and 506 of the GII.4 major capsid protein, VP1. [file jvi.01804-25-s0003.pdf]
