# Supplementary material for: Variable and conserved B cell epitopes of GII.4 human noroviruses
Source: J Virol. 2026 Jan 13;100(2):e01804-25. doi: 10.1128/jvi.01804-25 (PMC12911863; doi:10.1128/jvi.01804-25)
Supplement: Supplemental legends — Legends for Fig. S1 to S3. [file jvi.01804-25-s0004.docx]

**Supplementary Figure Legends**

**Fig. S1: Variability pattern of residues 297 and 372 of the GII.4 major capsid protein, VP1.** Patterns were calculated using amino acid combinations from 3108 VP1 sequences of GII.4 noroviruses as implemented elsewhere (1, 2).

**Fig. S2: GII.4 norovirus VP1 structures in complex with different broadly reactive monoclonal antibodies.** (A) Side and top view of the broadly reactive mouse antibody 5B18 (PDB: 3V7A) binding to VP1 protein (3). (B) Side and top view of the broadly reactive but non-blocking human antibody A1227 (PDB: 6N81) binding to VP1 protein (4). Side and lateral view of the human neutralizing antibodies (C) VX22 (PDB: 8VKX) and (D) NORO-320 (PDB: 7JIE) bind to a highly conserved region of the P1-P2 interphase (5, 6). The different monoclonal antibodies are shown in ribbon, the GII.4 antigenic sites are shown using the same colors as in Fig. 1. The structural model of norovirus VP1 dimer was acquired from the Protein Database (PDB: 7K6V) and the figures were rendered using UCSF Chimera (7, 8).

**Fig. S3: Variability pattern of residues 402, 403, 504, 506 of the GII.4 major capsid protein, VP1.** Patterns were calculated using amino acid combinations from 3108 VP1 sequences of GII.4 noroviruses as implemented elsewhere (1, 2). Only amino acid residue 504 presented variability that coincided with the emergence of the FarmingtonHills_2002 variant and subsequent variants.

**References**

1. Tohma K, Lepore CJ, Gao Y, Ford-Siltz LA, Parra GI. 2019. Population Genomics of GII.4 Noroviruses Reveal Complex Diversification and New Antigenic Sites Involved in the Emergence of Pandemic Strains. mBio 10.

2. Parra GI, Tohma K, Ford-Siltz LA, Eguino P, Kendra JA, Pilewski KA, Gao Y. 2023. Minimal Antigenic Evolution after a Decade of Norovirus GII.4 Sydney_2012 Circulation in Humans. J Virol 97:e0171622.

3. Hansman GS, Taylor DW, McLellan JS, Smith TJ, Georgiev I, Tame JR, Park SY, Yamazaki M, Gondaira F, Miki M, Katayama K, Murata K, Kwong PD. 2012. Structural basis for broad detection of genogroup II noroviruses by a monoclonal antibody that binds to a site occluded in the viral particle. J Virol 86:3635-46.

4. Lindesmith LC, McDaniel JR, Changela A, Verardi R, Kerr SA, Costantini V, Brewer-Jensen PD, Mallory ML, Voss WN, Boutz DR, Blazeck JJ, Ippolito GC, Vinje J, Kwong PD, Georgiou G, Baric RS. 2019. Sera Antibody Repertoire Analyses Reveal Mechanisms of Broad and Pandemic Strain Neutralizing Responses after Human Norovirus Vaccination. Immunity 50:1530-1541 e8.

5. Park J, Lindesmith LC, Olia AS, Costantini VP, Brewer-Jensen PD, Mallory ML, Kelley CE, Satterwhite E, Longo V, Tsybovsky Y, Stephens T, Marchioni J, Martins CA, Huang Y, Chaudhary R, Zweigart M, May SR, Reyes Y, Flitter B, Vinje J, Tucker SN, Ippolito GC, Lavinder JJ, Snijder J, Kwong PD, Georgiou G, Baric RS. 2025. Broadly neutralizing antibodies targeting pandemic GII.4 variants or seven GII genotypes of human norovirus. Sci Transl Med 17:eads8214.

6. Alvarado G, Salmen W, Ettayebi K, Hu L, Sankaran B, Estes MK, Venkataram Prasad BV, Crowe JE, Jr. 2021. Broadly cross-reactive human antibodies that inhibit genogroup I and II noroviruses. Nat Commun 12:4320.

7. Hu L, Salmen W, Chen R, Zhou Y, Neill F, Crowe JE, Jr., Atmar RL, Estes MK, Prasad BVV. 2022. Atomic structure of the predominant GII.4 human norovirus capsid reveals novel stability and plasticity. Nat Commun 13:1241.

8. Pettersen EF, Goddard TD, Huang CC, Couch GS, Greenblatt DM, Meng EC, Ferrin TE. 2004. UCSF Chimera--a visualization system for exploratory research and analysis. J Comput Chem 25:1605-12.
